# Identification of the lymph node metastasis-related automated breast volume scanning features for predicting axillary lymph node tumor burden of invasive breast cancer *via* a clinical prediction model

**DOI:** 10.3389/fendo.2022.881761

**Published:** 2022-08-05

**Authors:** Feng Zhao, Changjing Cai, Menghan Liu, Jidong Xiao

**Affiliations:** ^1^ Department of Cardiovascular Surgery, Xiangya Hospital, Central South University, Changsha, China; ^2^ National Clinical Research Center for Geriatric Disorders, Xiangya Hospital, Central South University, Changsha, China; ^3^ Department of Ultrasound, Third Xiangya Hospital, Central South University, Changsha, China; ^4^ Department of Oncology, Xiangya Hospital, Central South University, Changsha, China

**Keywords:** ABVS, ultrasound, breast cancer, ALND, SLNB

## Abstract

Breast cancer has become the malignant tumor with the highest incidence in women. Axillary lymph node dissection (ALND) is an effective method of maintaining regional control; however, it is associated with a significant risk of complications. Meanwhile, whether the patients need ALND or not is according to sentinel lymph node biopsy (SLNB). However, the false-negative results of SLNB had been reported. Automated breast volume scanning (ABVS) is a routine examination in breast cancer. A real-world cohort consisting of 245 breast cancer patients who underwent ABVS examination were enrolled, including 251 tumor lesions. The ABVS manifestations were analyzed with the SLNB results, and the ALND results for selecting the lymph node metastasis were related to ABVS features. Finally, a nomogram was used to construct a breast cancer axillary lymph node tumor burden prediction model. Breast cancer patients with a molecular subtype of luminal B type, a maximum lesion diameter of ≥5 cm, tumor invasion of the Cooper’s ligament, and tumor invasion of the nipple had heavy lymph node tumor burden. Molecular classification, tumor size, and Cooper’s ligament status were used to construct a clinical prediction model of axillary lymph node tumor burden. The consistency indexes (or AUC) of the training cohort and the validation cohort were 0.743 and 0.711, respectively, which was close to SLNB (0.768). The best cutoff value of the ABVS nomogram was 81.146 points. After combination with ABVS features and SLNB, the AUC of the prediction model was 0.889, and the best cutoff value was 178.965 points. The calibration curve showed that the constructed nomogram clinical prediction model and the real results were highly consistent. The clinical prediction model constructed using molecular classification, tumor size, and Cooper’s ligament status can effectively predict the probability of heavy axillary lymph node tumor burden, which can be the significant supplement to the SLNB. Therefore, this model may be used for individual decision-making in the diagnosis and treatments of breast cancer.

## Introduction

At present, breast cancer has become the malignant tumor with the highest incidence in women (1), and the onset of breast cancer has been occurring at younger and younger ages. Axillary lymph node dissection (ALND) is an effective method of maintaining regional control; however, it is associated with a significant risk of complications such as lymphedema, numbness, axillary web syndrome, and decreased upper-extremity range of motion (2, 3). The Z0011 trial conducted by the American College of Surgeons Oncology Group (ACOSOG) showed that if the postoperative treatments are standardized, patients with one or two positive lymph nodes in sentinel lymph node biopsy (SLNB) do not need an ALND (2, 4). Only breast cancer patients with three or more metastatic axillary lymph nodes are required to undergo surgical dissection. Therefore, Li et al. (5)proposed the concept of lymph node tumor burden, which defines fewer than three axillary lymph node metastases as a mild lymph node tumor burden, and three or more as a heavy lymph node tumor burden. The results of the ACOSOG Z0011 trial have changed the treatment of breast cancer. Studies have shown that the overall proportion of patients who met the Z0011 standard for parallel surgery has dropped from 34.0% to 22.7%, and there is a declining trend year by year (4, 6). Currently, lymphatic metastasis is mainly determined by SLNB; however, false-negative results (9.8%) had been reported (7).

Automated breast volume scanning (ABVS) is an emerging technology of breast ultrasound examination that can obtain images of multiple planes, including cross section, sagittal plane, and coronal plane. In addition, it can observe the lesions in real time, dynamically, continuously, and multi-sectionally, providing more information on the imaging manifestations of the lesions and the surrounding tissues of the lesions (8). Ultrasound is a common method for screening breast diseases, and the ultrasound manifestations of different molecular subtypes of breast cancer are slightly different, especially in ABVS technology (9). In addition, studies have shown that different molecular subtypes of breast cancer have different biological behaviors (10), different prognosis (11), and different distant metastasis statuses, i.e., the axillary lymph node metastasis status is different (12, 13). Therefore, if the relationship between the ABVS manifestations and the molecular subtype of the primary breast cancer lesion with the status of axillary lymph node metastasis can be ascertained, more imaging evidence for assessing the status of the lymph node metastasis can be provided.

Therefore, we initiated a real-world analysis. First, we performed some analyses of the clinical features, ABVS features, and lymph node tumor burden. Then, the features related to the lymph node tumor burden were selected. Finally, a clinical prediction model of lymph node tumor burden was developed. Our work indicated there are strong links between ABVS features and lymph node tumor burden, and the clinical prediction model can be the significant supplement to the SLNB, and this model may be used for individual decision-making.

## Materials and methods

### Xiangya real-world cohort patients

The patients who underwent ABVS examination in the Department of Ultrasound of Third Xiangya Hospital of Central South University and were confirmed to have breast cancer by postoperative pathological examination from June 2017 to June 2019 were included. There was a total of 245 patients and 251 tumor lesions. The patients were screened according to the inclusion criteria and exclusion criteria. This study was approved by the ethics committee of Third Xiangya Hospital of Central South University.

The inclusion criteria were (1) preoperative ABVS examination in our hospital and postoperative pathological confirmation of breast cancer and (2) complete clinical and pathological data.

The exclusion criteria were as follows (1): not newly diagnosed with breast cancer (2); the patients without the ABVS results before neoadjuvant chemotherapy (chemotherapy before surgery); and (3) the clinical data and pathological results were incomplete (4); the patients with poor-quality ABVS images: the scanning operation is not standardized— the breast gland scanning is incomplete, the scanning depth is too large or too small, and the gain is too large or too small, the gray-scale setting is based on fat tissue, and the fat lobules are medium gray, not black—and artifacts: the probe does not fit well with the patient’s skin, causing artifacts, and the glands are not flattened; the posterior echo attenuation of the image generated by the wrinkles in the nipple and areola area, and coupling agent solidification, small bubbles.

The patients with the relative contraindication for ABVS examination were described as the following: there is no absolute contraindication, but it is recommended to use it with caution or check it after full communication with the patient in the following cases—in the middle and late trimesters of pregnancy, lactation, acute mastitis, great pain of breast, breast prosthesis, and breast skin ulceration.

### ABVS examination

A Siemens ACUSON S2000 ABVS acquisition system was used for image acquisition for all of the selected subjects; the probe model was 14L5BV, the frequency was 5.0–12.0 MHz, and the maximum scan volume was 154 mm × 168 mm × 60 mm. The patient was in a supine position with both hands raised over the head to fully expose the breasts on both sides. The mechanical arm was adjusted so that the probe could exert proper pressure to contact the breast without causing patient discomfort. The settings of the instrument were preset according to the size of the patient’s breasts. Then the machine scanned the median, lateral, and medial positions of the breast sequentially and, when necessary, scanned other planes. After the scan was completed, the position of the nipple was marked, and the images were uploaded to the image processing workstation for image reconstruction. If a mass was identified, the image features of the mass on the ABVS images were extracted, including tumor size (≤2 cm/2–5 cm/≥5 cm). Clinically, the TNM staging method is used for clinical staging of breast cancer, where T represents the size of the tumor, N represents lymph node invasion, and M represents distant metastasis. T1 indicates that the maximum diameter of the lesion is ≤2 cm, T2 indicates that the maximum diameter of the lesion is 2–5 cm, and T3 indicates that the maximum diameter of the lesion is ≥5 cm, which is the current T staging standard and also the size grouping method used in this study. The use of tumor size to determine the degree of breast cancer malignancy and the range of invasiveness has been recognized. Shape (regular/irregular), margin (circumscribed/angular/microlobulated/spiculated), orientation (parallel/non-parallel), echo pattern (hypoechoic/mixed solid echo), posterior acoustic pattern (enhanced/shadow/no change), retraction phenomenon (present/absent), acoustic halo (present/absent), microcalcification (present/absent: microcalcifications were observed as echogenic dots within the mass or as a dilated duct on the ABVS images (14)), invasion of Cooper’s ligament (present/absent: Cooper’s ligaments were considered shortened, thickened, pulled, and straightened, when there were hyperechogenic lines near the mass, radiating toward the skin and thus differing from other parts of normal breast tissue. Cooper’s ligaments were considered normal if this feature was absent (15)), and BI-RADS (breast imaging reporting and data system) classification (class 3/4a/4b/4c/5) were determined. Two doctors independently evaluated all of the acoustic image characteristics with intermediate or higher titles. Disagreements were resolved by a third doctor with a senior title. According to the study of Eda et al., for mass lesions, malignant features include irregular margin, irregular shape, non-parallel growth, peripheral hyperechoic halo, posterior acoustic pattern attenuation, and microcalcification. In addition, one malignant sign is categorized as class 4a, two malignant signs as class 4b, three as class 4c, and more than three as class 5 (16).

### Determination of surrogate molecular subtypes

According to the St. Gallen consensus and ASCO/CAP (American Society of Clinical Oncology/College of American Pathologists) guidelines, if the stained cells exceed 1% of the total number of cells, the patient is considered PR- (progesterone receptor) and ER- (estrogen receptor) positive; if the number of stained cells is less than 1% of the total number of cells, the patient is considered PR- and ER-negative (17, 18). The Ki-67 proliferation index is determined by the percentage of the number of stained cells in the total number of tumor cells, with 20% as the cutoff value (<20% is considered low proliferation, and ≥20% is considered high proliferation (19, 20)). The *HER2* (human epidermal growth factor receptor 2) gene was detected using immunohistochemistry (IHC), and the IHC results were scored as 0, 1+, 2+, and 3+ according to the standards. The 2+ specimens were further examined using fluorescence *in situ* hybridization (FISH), and the result was used as a basis for further judgment of the amplification of the *HER2* gene. *HER2*-positive cases included IHC 3+ and FISH-positive individuals of IHC 2+ cases, and *HER2*-negative cases included IHC 0, 1+, and FISH-negative individuals of IHC 2+ cases (21). Surrogate molecular subtypes were as follows (1): luminal A: ER or PR positive, and Ki-67 of less than 20% (2); luminal B: with ER or PR positive, and Ki-67 of 20% or greater (3); HER2: ER negative, PR negative, and HER2 positive; and (4) triple negative: ER negative, PR negative, and HER2 negative.

### Grouping criteria for axillary lymph node tumor burden

According to the ACOSOG Z0011 trial results, if follow-up tumor surgery and postoperative comprehensive treatment are standardized, patients with two or fewer positive SLNB do not need to undergo ALND (22). Therefore, sentinel/axillary lymph node metastasis ≥3 is defined as heavy lymph node tumor burden, and sentinel/axillary lymph node metastasis <3 is defined as mild lymph node tumor burden (5).

### Statistics

Using R software (RStudio 1.2) and SPSS 25.0 software, the relationship between the ABVS manifestations of different molecular subtypes of breast cancer and lymph node tumor burden was explored through logistic univariate and multivariate regression risk factor analyses. Using SPSS 25.0 software, the chi-square test or Fisher’s exact test was performed to analyze the difference between the mild axillary lymph node tumor burden group and the heavy axillary lymph node tumor burden group. (1. For all theoretical numbers T ≥5 and total sample size n ≥ 40, the Pearson chi-square test was used; 2. If theoretical number T < 5 but ≥ 1, and n ≥ 40, a continuity correction chi-square test was performed; 3. If the theoretical number T <1 or n <40, a Fisher’s test was used.) Finally, based on the logistic regression analysis results, R software was used to construct a nomogram model for breast cancer axillary lymph node tumor burden prediction. MedCalc software (18.2) was used to graph the ROC curve of axillary lymph node tumor burden detected using SLNB and calculate the area under the curve.

## Results

### Clinical characteristics and pathological data of the study subjects

According to the inclusion and exclusion criteria, a total of 245 eligible patients were screened, and a total of 251 lesions were included in the statistical analysis (**Table S1**).

### Relationship between ABVS manifestations of different molecular subtypes of breast lesions and different levels of axillary lymph node tumor burden

#### Difference of lymph node tumor burden in the ABVS manifestations and molecular subtypes of different breast lesions

A total of 251 breast lesions were included. Among the 144 lesions that underwent SLNB, the size, orientation, echo pattern, shape, margin, posterior acoustic pattern, presence of acoustic halo, presence of microcalcification, presence of retraction phenomenon, lesion type, and invasion of the Cooper’s ligament were not significantly different between the mild lymph node tumor burden group and the heavy lymph node tumor burden group (P > 0.05).

In the 178 cases of lesions that performed ALND, the shape, margin, orientation, echo pattern, posterior acoustic pattern, retraction phenomenon, and microcalcification were not significantly different between the mild lymph node tumor burden group and the heavy lymph node tumor burden group (P> 0.05). However, the tumor size in the heavy lymph node burden group was larger than in the mild lymph node tumor burden group with statistical significance (χ^2^ = 7.594, P = 0.022). The incidence of acoustic halo in the heavy lymph node tumor burden group was higher than in the mild lymph node tumor burden group with statistical significance (χ^2^ = 5.753, P = 0.016). The Cooper’s ligament invasion proportion in the heavy lymph node tumor burden group was higher than in the mild lymph node tumor burden group with statistical significance (χ^2^ = 11.992, P = 0.001).

We did not detect a significant difference in the sentinel lymph node tumor burden in the analysis of different molecular subtypes. However, in the analysis of axillary lymph node tumor burden, we found that in the luminal A type, *HER-2* overexpression type, and triple-negative breast cancer, the proportion of patients with a mild lymph node tumor burden was significantly higher than that of patients with a heavy lymph node tumor burden. In contrast, in luminal B breast cancer, the proportion of patients with heavy lymph node tumor burden was significantly higher than that of patients with mild lymph node tumor burden (72.549% vs. 57.480%, χ^2^ = 8.050, P = 0.046). Our results suggest that molecular classification is an important factor affecting the axillary lymph node tumor burden. Therefore, we further analyzed the subgroups of different molecular subtypes (**Table 1**).

**Table 1:** Difference of lymph node tumor burden in the ABVS features and molecular subtypes of different breast lesions.

ABVS features and molecular subtype	Sentinel lymph node tumor burden			Axillary lymph node tumor burden		
	Mild	Heavy	P	Mild	Heavy	P
Tumor size						
≥5 cm	8 (5.970%)	2 (20.000%)	0.155	6 (4.724%)	8 (15.686%)	0.022
2–5 cm	74 (55.224%)	6 (60.000%)		71 (55.906%)	30 (58.824%)	
≤2 cm	52 (38.806%)	2 (20.000%)		50 (39.370%)	13 (25.490%)	
Orientation						
Not parallel	45 (33.582%)	2 (20.000%)	0.499	48 (37.795%)	17 (33.333%)	0.699
Parallel	89 (66.418%)	8 (80.000%)		79 (62.205%)	34 (66.667%)	
Shape						
Regular	17 (12.687%)	0 (0.000%)	0.609	12 (9.449%)	3 (5.882%)	0.560
Irregular	117 (87.313%)	10 (100.000%)		115 (90.551%)	48 (94.118%)	
Echo pattern						
Hypoechoic	119 (88.806%)	10 (100.000%)	0.600	118 (92.913%)	49 (96.078%)	0.731
Mixed solid echo	15 (11.194%)	0 (0.000%)		9 (7.087%)	2 (3.922%)	
Margin						
Circumscribed	10 (7.462%)	0 (0.000%)	0.116	3 (2.362%)	0 (0.000%)	0.085
Angular	51 (38.060%)	4 (40.000%)		46 (36.220%)	15 (29.412%)	
Microlobulated	18 (13.433%)	0 (0.000%)		20 (15.748%)	3 (%5.882)	
Spiculated	55 (41.045%)	6 (60.000%)		58 (45.669%)	33 (64.706%)	
Posterior acoustic pattern						
Enhancement	22 (16.418%)	1 (10.000%)	1.000	21 (16.535%)	5 (9.804%)	0.103
Shadow	22 (16.418%)	2 (20.000%)		22 (17.323%)	16 (31.373%)	
No change	90 (67.164%)	7 (70.000%)		84 (66.142%)	30 (58.824%)	
Microcalcifications						
Present	52 (38.806%)	3 (30.000%)	0.742	59 (46.457%)	18 (35.294%)	0.233
Absent	82 (61.194%)	7 (70.000%)		68 (53.543%)	33 (64.706%)	
Acoustic halo						
Present	28 (20.896%)	2 (20.000%)	1.000	22 (17.323%)	18 (35.294%)	0.016
Absent	106 (79.104%)	8 (80.000%)		105 (82.677%)	33 (64.706%)	
Retraction phenomenon						
Present	17 (12.687%)	1 (10.000%)	1.000	25 (19.685%)	10 (19.608%)	1.000
Absent	117 (87.313%)	9 (90.000%)		102 (80.315%)	41 (80.392%)	
Invasion of Cooper’s ligament						
Yes	29 (21.642%)	3 (30.000%)	0.693	37 (29.134%)	29 (56.863%)	0.001
No	105 (78.358%)	7 (70.000%)		90 (70.866%)	22 (43.137%)	
BI-RADS						
3	11 (8.209%)	0 (0.000%)	0.882	0 (0.000%)	0 (0.000%)	0.068
4a	25 (18.657%)	2 (20.000%)		14 (11.024%)	5 (9.804%)	
4b	44 (32.836%)	3 (30.000%)		48 (37.795%)	10 (19.608%)	
4c	18 (13.433%)	2 (20.000%)		24 (18.898%)	10 (19.608%)	
5	36 (26.865%)	3 (30.000%)		41 (32.283%)	26 (50.980%)	
Molecular subtype						
Luminal A	41 (30.597%)	1 (10.000%)	0.226	26 (14.173%)	4 (7.843%)	0.046
Luminal B	60 (44.776%)	8 (80.000%)		65 (57.480%)	37 (72.549%)	
HER-2	17 (12.687%)	1 (10.000%)		20 (15.748%)	4 (7.843%)	
Triple negative	16 (11.940%)	0 (0.000%)		16 (12.598%)	6 (11.765%)	

#### Differences of lymph node tumor burden of luminal A-type breast cancer regarding different ABVS manifestations

In 44 cases of luminal A-type lesions, the lymph node tumor burden was not significantly different with respect to tumor size, shape, margin, orientation, echo pattern, posterior acoustic pattern, retraction phenomenon, microcalcification, invasion of the Cooper’s ligament, and BI-RADS classification (P > 0.05). The incidence of acoustic halo in the heavy lymph node tumor burden group was higher than in the mild lymph node tumor burden group with statistical significance (χ^2^ = 8.734, P = 0.003) (**Table 2**).

**Table 2:** Differences of lymph node tumor burden of luminal A-type breast cancer regarding different ABVS feathers.

ABVS feathers	Sentinel lymph node tumor burden			Axillary lymph node tumor burden		
	Mild	Heavy	P	Mild	Heavy	P
Tumor size						
≥5 cm	4 (9.756%)	1 (100.000%)	0.119	1 (3.846%)	1 (25.000%)	0.328
2–5 cm	19 (46.341%)	0 (0.000%)		12 (46.154%)	1 (25.000%)	
≤2 cm	18 (43.902%)	0 (0.000%)		13 (50.000%)	2 (50.000%)	
Orientation						
~	13 (31.707%)	0 (0.000%)	1.000	8 (30.769%)	0 (0.000%)	0.550
Parallel	28 (68.293%)	1 (100.000%)		18 (69.231%)	4 (100.000%)	
Shape						
Regular	4 (9.756%)	0 (0.000%)	1.000	0 (0.000%)	0 (0.000%)	1.000
Irregular	37 (90.244%)	1 (100.000%)		26 (100.000%)	4 (100.000%)	
Echo pattern						
Hypoechoic	35 (85.366%)	1 (100.000%)	1.000	24 (92.308%)	3 (75.000%)	0.360
Mixed solid echo	6 (14.634%)	0 (0.000%)		2 (7.692%)	1 (25.000%)	
Margin						
Circumscribed	5 (12.195%)	0 (0.000%)	1.000	1 (3.846%)	0 (0.000%)	0.154
Angular	16 (39.024%)	1 (100.000%)		7 (26.923%)	2 (50.000%)	
Microlobulated	3 (7.317%)	0 (0.000%)		1 (3.846%)	1 (25.000%)	
Spiculated	17 (41.463%)	0 (0.000%)		17 (65.385%)	1 (25.000%)	
Posterior acoustic pattern						
Enhancement	6 (14.634%)	0 (0.000%)	1.000	2 (7.692%)	1 (25.000%)	0.452
Shadow	7 (17.073%)	0 (0.000%)		5 (19.231%)	0 (0.000%)	
No change	28 (68.293%)	1 (100.000%)		19 (73.077%)	3 (75.000%)	
Microcalcifications						
Present	11 (26.829%)	0 (0.000%)	1.000	12 (46.154%)	0 (0.000%)	0.130
Absent	30 (70.171%)	1 (100.000%)		14 (53.846%)	4 (100.000%)	
Acoustic halo						
Present	6 (14.634%)	1 (100.000%)	0.167	4 (15.385%)	4 (100.000%)	0.003
Absent	35 (85.366%)	0 (0.000%)		22 (84.615%)	0 (0.000%)	
Retraction phenomenon						
Present	4 (9.756%)	0 (0.000%)	1.000	7 (26.923%)	1 (25.000%)	1.000
Absent	37 (90.244%)	1 (100.000%)		19 (73.077%)	3 (75.000%)	
Invasion of Cooper’s ligament						
Yes	9 (21.951%)	0 (0.000%)	1.000	10 (38.462%)	2 (50.000%)	1.000
No	32 (78.049%)	1 (100.000%)		16 (61.538%)	2 (50.000%)	
BI-RADS						
3	7 (17.073%)	0 (0.000%)	1.000	0 (0.000%)	0 (0.000%)	0.220
4a	8 (19.512%)	0 (0.000%)		0 (0.000%)	1 (25.000%)	
4b	12 (29.268%)	1 (100.000%)		14 (53.846%)	2 (50.000%)	
4c	4 (9.756%)	0 (0.000%)		4 (15.385%)	0 (0.000%)	
5	10 (24.390%)	0 (0.000%)		8 (30.769%)	1 (25.000%)	

#### Differences of lymph node tumor burden of luminal B-type breast cancer regarding different ABVS manifestations

A total of 138 cases of luminal B-type breast lesions were included. Among the 73 lesions that underwent SLNB, the lymph node tumor burden was not significantly different with respect to tumor size, shape, margin, orientation, echo pattern, posterior acoustic pattern, retraction phenomenon, acoustic halo, microcalcification, invasion of the Cooper’s ligament, and BI-RADS classification (P > 0.05). In the 113 cases of lesions that underwent ALND, the lymph node tumor burden was not significantly different concerning tumor size, shape, margin, orientation, echo pattern, posterior acoustic pattern, retraction phenomenon, acoustic halo, microcalcification, invasion of the Cooper’s ligament, and BI-RADS classification. The proportion of Cooper’s ligament invasion in the heavy lymph node tumor burden group was higher than in the mild lymph node tumor burden group with statistical significance (χ^2^ = 7.749, P= 0.005) (**Table 3**).

**Table 3:** Differences of lymph node tumor burden of luminal B-type breast cancer regarding different ABVS features.

ABVS features	Sentinel lymph node tumor burden			Axillary lymph node tumor burden		
	Mild	Heavy	P	Mild	Heavy	P
Tumor size						
≥5 cm	3 (5.000%)	1 (12.500%)	0.173	3 (4.615%)	5 (13.514%)	0.150
2–5 cm	34 (56.667%)	6 (75.000%)		33 (50.769%)	21 (56.757%)	
≤2 cm	23 (38.333%)	1 (12.500%)		29 (44.615%)	11 (29.730%)	
Orientation						
Not parallel	22 (36.667%)	1 (12.500%)	0.250	29 (44.615%)	12 (32.432%)	0.295
Parallel	38 (63.333%)	7 (87.500%)		36 (55.385%)	25 (67.568%)	
Shape						
Regular	7 (11.667%)	0 (0.000%)	0.587	3 (4.615%)	3 (8.108%)	0.665
Irregular	53 (88.333%)	8 (100.000%)		62 (95.385%)	34 (91.892%)	
Echo pattern						
Hypoechoic	57 (95.000%)	8 (100.000%)	1.000	63 (96.923%)	36 (97.297%)	1.000
Mixed solid echo	3 (5.000%)	0 (0.000%)		2 (3.077%)	1 (2.703%)	
Margin						
Circumscribed	4 (6.667%)	0 (0.000%)	0.301	1 (1.538%)	0 (0.000%)	0.133
Angular	24 (40.000%)	2 (25.000%)		24 (36.923%)	10 (27.027%)	
Microlobulated	10 (16.667%)	0 (0.000%)		10 (15.385%)	2 (5.405%)	
Spiculated	22 (36.667%)	6 (75.000%)		30 (46.154%)	25 (67.568%)	
Posterior acoustic pattern						
Enhancement	8 (13.333%)	0 (0.000%)	0.723	8 (0.123%)	3 (8.108%)	0.501
Shadow	12 (20.000%)	2 (25.000%)		14 (21.538%)	12 (32.432%)	
No change	40 (66.667%)	6 (75.000%)		43 (66.154%)	22 (59.459%)	
Microcalcifications						
Present	33 (55.000%)	3 (37.500%)	0.461	33 (50.769%)	18 (48.649%)	1.000
Absent	27 (45.000%)	5 (62.5%)		32 (49.231%)	19 (51.351%)	
Acoustic halo						
Present	17 (28.333%)	1 (12.500%)	0.671	13 (20.000%)	13 (35.135%)	0.104
Absent	43 (71.667%)	7 (87.500%)		52 (80.000%)	24 (64.865%)	
Retraction phenomenon						
Present	12 (20.000%)	1 (12.500%)	1.000	15 (23.076%)	9 (24.324%)	1.000
Absent	48 (80.000%)	7 (87.500%)		50 (76.923%)	28 (75.676%)	
Invasion of Cooper’s ligament						
Yes	18 (30.000%)	3 (37.500%)	0.695	19 (29.231%)	22 (59.459%)	0.005
No	42 (70.000%)	5 (62.500%)		46 (70.769%)	15 (40.541%)	
BI-RADS						
3	2 (3.333%)	0 (0.000%)	0.803	0 (0.000%)	0 (0.000%)	0.126
4a	9 (15.000%)	2 (25.000%)		9 (13.846%)	2 (5.405%)	
4b	17 (28.333%)	1 (12.500%)		18 (27.692%)	5 (13.514%)	
4c	13 (21.667%)	2 (25.000%)		14 (21.538%)	9 (24.324%)	
5	19 (31.667%)	3 (37.500%)		24 (36.923%)	21 (56.757%)	

#### Differences of lymph node tumor burden of HER-2 overexpression type breast cancer regarding different ABVS manifestations

A total of 35 cases of *HER-2* overexpression breast lesions were included. Among the 18 lesions that underwent SLNB, the lymph node tumor burden was not significantly different with respect to tumor size, shape, margin, orientation, echo pattern, posterior acoustic pattern, retraction phenomenon, acoustic halo, microcalcification, invasion of the cooper’s ligament, and BI-RADS classification (P > 0.05). In the 24 cases of lesions that underwent ALND, the lymph node tumor burden was not significantly different with respect to tumor size, shape, margin, orientation, echo pattern, posterior acoustic pattern, retraction phenomenon, acoustic halo, microcalcification, invasion of the Cooper’s ligament, and BI-RADS classification (P > 0.05). The proportion of posterior acoustic pattern in the heavy lymph node tumor burden group was higher than in the mild lymph node tumor burden group with statistical significance (χ^2^ = 6.900, P = 0.032) (**Table 4**).

**Table 4:** Differences of lymph node tumor burden of HER-2 overexpression type breast cancer regarding different ABVS features.

ABVS features	Sentinel lymph node tumor burden			Axillary lymph node tumor burden		
	Mild	Heavy	P	Mild	Heavy	P
Tumor size						
≥5 cm	1 (5.882%)	0 (0.000%)	0.389	1 (5.000%)	1 (25.000%)	0.405
2–5 cm	11 (64.706%)	0 (0.000%)		15 (75.000%)	3 (75.000%)	
≤2 cm	5 (29.412%)	1 (100.000%)		4 (20.000%)	0 (0.000%)	
Orientation						
Not parallel	3 (17.647%)	0 (0.000%)	1.000	5 (25.000%)	3 (75.000%)	0.091
Parallel	14 (82.353%)	1 (100.000%)		15 (75.000%)	1 (25.000%)	
Shape						
Regular	1 (5.882%)	0 (0.000%)	1.000	3 (15.000%)	0 (0.000%)	1.000
Irregular	16 (94.118%)	1 (100.000%)		17 (85.000%)	4 (100.000%)	
Echo pattern						
Hypoechoic	15 (88.235%)	1 (100.000%)	1.000	17 (85.000%)	4 (100.000%)	1.000
Mixed solid echo	2 (11.765%)	0 (0.000%)		3 (15.000%)	0 (0.000%)	
Margin						
Circumscribed	1 (5.882%)	0 (0.000%)	0.389	0 (0.000%)	0 (0.000%)	0.135
Angular	4 (23.529%)	1 (100.000%)		8 (40.000%)	0 (0.000%)	
Microlobulated	1 (5.882%)	0 (0.000%)		4 (20.000%)	0 (0.000%)	
Spiculated	11 (64.706%)	0 (0.000%)		8 (40.000%)	4 (100.000%)	
Posterior acoustic pattern						
Enhancement	2 (11.765%)	0 (0.000%)	0.278	7 (35.000%)	1 (25.000%)	0.032
Shadow	2 (11.765%)	1 (100.000%)		3 (15.000%)	3 (75.000%)	
No change	13 (76.471%)	0 (0.000%)		10 (50.000%)	0 (0.000%)	
Microcalcifications						
Present	6 (35.294%)	0 (0.000%)	1.000	10 (50.000%)	0 (0.000%)	0.114
Absent	11(64.706%)	1 (100.000%)		10 (50.000%)	4 (100.000%)	
Acoustic halo						
Present	2 (11.765%)	0 (0.000%)	1.000	1 (5.000%)	0 (0.000%)	1.000
Absent	15 (88.235%)	1 (100.000%)		19 (95.000%)	4 (100.000%)	
Retraction phenomenon						
Present	0 (0.000%)	0 (0.000%)	1.000	1 (5.000%)	0 (0.000%)	1.000
Absent	17 (100.00%)	1 (100.000%)		19 (95.000%)	4 (100.000%)	
Invasion of Cooper’s ligament						
Yes	2 (11.765%)	0 (0.000%)	1.000	4 (20.000%)	1 (25.000%)	1.000
No	15 (88.235%)	1 (100.000%)		16 (80.000%)	3 (75.000%)	
BI-RADS						
3	1 (5.882%)	0 (0.000%)	0.645	0 (0.000%)	0 (0.000%)	0.223
4a	4 (23.529%)	0 (0.000%)		0 (0.000%)	0 (0.000%)	
4b	6 (35.294%)	1 (100.000%)		9 (45.000%)	0 (0.000%)	
4c	0 (0.000%)	0 (0.000%)		2 (10.000%)	1 (25.000%)	
5	6 (35.294%)	0 (0.000%)		9 (45.000%)	3 (75.000%)	

#### Differences of lymph node tumor burden of triple-negative type breast cancer regarding different ABVS manifestations

A total of 34 cases of triple-negative breast lesions were enrolled, of which 16 cases underwent SLNB and 22 cases underwent ALND. According to the results of SLNB, no patients were with heavy lymph node tumor burden. In the 22 cases of lesions that underwent ALND, the lymph node tumor burden was not significantly different concerning tumor size, shape, margin, orientation, echo pattern, posterior acoustic pattern, retraction phenomenon, acoustic halo, microcalcification, Cooper’s ligament invasion, and BI-RADS classification (P > 0.05). The BI-RADS classification of the heavy lymph node tumor burden group was higher than that of the mild lymphoid tumor burden group with statistical significance (χ^2^ = 13.387, P = 0.004) (**Table 5**).

**Table 5:** Differences of lymph node tumor burden of triple-negative type breast cancer regarding different ABVS features.

ABVS features	Sentinel lymph node tumor burden			Axillary lymph node tumor burden		
	Mild	Heavy	P	Mild	Heavy	P
Tumor size						
≥5 cm	0 (0.000%)	0 (0.000%)	/	1 (6.250%)	1 (16.667%)	0.424
2–5 cm	10 (62.500)	0 (0.000%)		11 (68.750%)	5 (83.333%)	
≤2 cm	6 (37.500%)	0 (0.000%)		4 (25.000%)	0 (0.000%)	
Orientation						
Not parallel	7 (43.750%)	0 (0.000%)	/	6 (37.500%)	2 (33.333%)	1.000
Parallel	9 (56.250%)	0 (0.000%)		10 (62.500%)	4 (66.667%)	
Shape						
Regular	5 (31.250%)	0 (0.000%)	/	6 (37.500%)	0 (0.000%)	0.133
Irregular	11 (68.750%)	0 (0.000%)		10 (62.500%)	6 (100.000%)	
Margin						
Circumscribed	0 (0.000%)	0 (0.000%)	/	1 (6.250%)	0 (0.000%)	0.340
Angular	7 (43.750%)	0 (0.000%)		7 (43.750%)	3 (50.000%)	
Microlobulated	4 (25.000%)	0 (0.000%)		5 (31.250%)	0 (0.000%)	
Spiculated	5 (31.250%)	0 (0.000%)		3 (18.750%)	3 (50.000%)	
Echo pattern						
Hypoechoic	12 (75.000%)	0 (0.000%)	/	14 (87.500%)	6 (100.000%)	1.000
Mixed solid echo	4 (25.000%)	0 (0.000%)		2 (12.500%)	0 (0.000%)	
Posterior acoustic pattern						
Enhancement	6 (37.500%)	0 (0.000%)	/	4 (25.000%)	0 (0.000%)	0.183
Shadow	1 (6.250%)	0 (0.000%)		0 (0.000%)	1 (16.667%)	
No change	9 (56.250%)	0 (0.000%)		12 (75.000%)	5 (83.333%)	
Microcalcifications						
Present	2 (12.500%)	0 (0.000%)	/	4 (25.000%)	0 (0.000%)	0.541
Absent	14 (87.500%)	0 (0.000%)		12 (75.000)	6 (100.000%)	
Acoustic halo						
Present	3 (18.750%)	0 (0.000%)	/	4 (25.000%)	1 (16.667%)	1.000
Absent	13 (81.250%)	0 (0.000%)		12 (75.000%)	5 (83.333%)	
Retraction phenomenon						
Present	1 (6.250%)	0 (0.000%)	/	2 (12.500%)	0 (0.000%)	1.000
Absent	15 (93.750%)	0 (0.000%)		14 (87.500%)	6 (100.000%)	
Invasion of Cooper’s ligament						
Yes	0 (0.000%)	0 (0.000%)	/	4 (25.000%)	4 (66.667%)	0.137
No	16 (100.000%)	0 (0.000%)		12 (75.000%)	2 (33.333%)	
BI-RADS						
3	1 (6.250%)	0 (0.000%)	/	0 (0.000%)	0 (0.000%)	0.004
4a	4 (25.000%)	0 (0.000%)		5 (31.250%)	1 (16.667%)	
4b	9 (55.250%)	0 (0.000%)		7 (43.750%)	1 (16.667%)	
4c	1 (6.250%)	0 (0.000%)		4 (25.000%)	0 (0.000%)	
5	1 (6.250%)	0 (0.000%)		0 (0.000%)	4 (66.667%)	

### Relationship between ABVS manifestations and different levels of axillary lymph node tumor burden

#### Relationship between breast cancer ABVS manifestations and clinical features with sentinel lymph node tumor burden

To better explore the relationship between the clinical features of breast cancer and sentinel lymph node tumor burden, which can rule out the influence of other factors, including age, molecular subtype, Ki-67, neoadjuvant chemotherapy, menopause, and tumor site, a univariate logistic regression analysis was performed. The results showed that none of the above factors was statistically significant (P > 0.05) (**Table S2**).

ABVS manifestations, tumor size, shape, margin, orientation, echo Pattern, posterior acoustic pattern, retraction phenomenon, acoustic halo, microcalcification, Cooper’s ligament invasion, and BI-RADS classification were included in an univariate logistic regression analysis. The results showed that the maximum lesion diameter of ≥5 cm significantly influenced the aggravation of sentinel lymph node tumor burden. At the same time, the difference was not statistically significant (OR = 6.500, 95% CI 0.701–60.974, P= 0.080; **Table S3**). Therefore, indicators with a P-value <0.05 can be included in the multivariate logistic regression analysis. However, because none of the univariate logistic regression analysis results of this study was statistically significant, no indicator could be included in multivariate logistic regression analysis in this study.

#### Relationship between breast cancer ABVS manifestations and clinical features with axillary lymph node tumor burden

To better explore the relationship between the clinical features of breast cancer and axillary lymph node tumor burden, which can rule out the influence of other factors, including age, molecular subtype, Ki-67, neoadjuvant chemotherapy, menopause, and tumor site, an univariate logistic regression analysis was performed. Neoadjuvant chemotherapy was a risk factor of heavy axillary lymph node tumor burden (OR = 4.181, 95% CI 1.509–12.202, P = 0.006), and nipple invasion significantly increased the risk of heavy axillary lymph node tumor burden (OR = 6.793, 95% CI Is 1.411–48.598, P = 0.025) (**Table S4**).

Studies have shown that different molecular subtypes of breast cancer have different prognoses (11). Neoadjuvant chemotherapy can downgrade the clinical stage of breast cancer patients and has different responses in different molecular subtypes (23), suggesting that it may affect postoperative lymph node tumor burden. Therefore, we included indicators with a P-value <0.05 and clinical significance in the multivariate logistic regression analysis. Molecular classification and Ki-67 were included in the multivariate logistic regression analysis together with neoadjuvant chemotherapy and nipple invasion. The results showed that the molecular subtype of the luminal B type (OR = 7.766, 95% CI 2.022–43.649, P = 0.008) was an independent risk factor of heavy axillary lymph node tumor burden; in addition, neoadjuvant chemotherapy (OR = 6.657, 95% CI 2.017–24.579, P = 0.003) was also one of the risk factors. We conducted a literature review and data analysis and found a false-positive result, which will be discussed in the discussion section (**Table 6**).

**Table 6:** Multivariate-logistic regression analysis of the clinical features and axillary lymph node tumor burden.

Variable	OR (95% CI)	P
Molecular subtype		
Luminal A	1.666 (0.292-10.933)	0.570
Luminal B	7.766 (2.022-43.649)	0.008
Triple negative	3.288 (0.645-20.811)	0.169
HER-2	1.000	
Ki-67		
≥20%	1.705 (0.427-9.279)	0.483
<20%	1.000	
Neoadjuvant chemotherapy		
Yes	6.657 (2.017-24.57)	0.003	
No	1.000		
Nipple invasion			
Present	14.147 (2.186-133.948)	0.009	
Absent	1.000		

Tumor size, shape, margin, orientation, echo pattern, posterior acoustic pattern, retraction phenomenon, acoustic halo, microcalcification, Cooper’s ligament invasion, and BI-RADS classification were included in univariate logistic regression analysis. The results showed that the maximum lesion diameter of ≥5 cm significantly increased the risk of heavy axillary lymph node tumor burden (OR = 5.128, 95% CI 1.530–18.232, P = 0.009), as well as a lesion with acoustic halo (OR = 2.603, 95% CI 1.242–5.446, P = 0.011) and invasion of the Cooper’s ligament (OR = 3.206, 95% CI 1.645–6.353, P = 0.001) (**Table S5**).

The multivariate logistic regression analysis included indicators with a P-value <0.05. To exclude the influence of other factors, the significant factors in the multivariate logistic regression analysis of the relationship between clinical features and axillary lymph node tumor burden were also included in the multivariate logistic regression analysis. The included factors were molecular subtype, neoadjuvant chemotherapy, lesion size, acoustic halo, posterior acoustic pattern, and Cooper’s ligament invasion. The results showed that the molecular subtype of luminal B type (OR = 4.405, 95% CI was 1.194–20.368, P = 0.037), maximum lesion diameter of ≥5 cm (OR = 8.734, 95% CI was 2.156–38.796, P = 0.003), and tumor invasion of Cooper’s ligament (OR = 3.295, 95% CI 1.529–7.303, P = 0.004) were independent influence factors of heavy axillary lymph node tumor burden. Moreover, similar to the above analysis, neoadjuvant chemotherapy (OR = 6.951, 95% CI 2.133–25.144, P = 0.002 was also one of the risk factors. We conducted a literature review and data analysis and found that this is a false-positive result, which will be discussed in the discussion section (**Table 7**).

**Table 7:** Multivariate-logistic regression analysis of the ABVS features and axillary lymph node tumor burden.

Variable	OR (95% CI)	P
Molecular subtype		
Luminal A	0.939 (0.153-5.851)	0.945
Luminal B	4.405 (1.194-20.368)	0.037
Triple negative	2.028 (0.381-11.803)	0.412
HER-2	1.000	
Neoadjuvant chemotherapy		
Yes	6.951 (2.133-25.144)	0.002
No	1.000	
Tumor size		
≥5 cm	8.734 (2.156-38.796)	0.003
2–5 cm	1.491 (0.629-3.648)	0.370
≤2 cm	1.000	
Acoustic halo		
Present	2.205 (0.910-5.358)	0.078
Absent	1.000	
Invasion of Cooper’s ligament		
Yes	3.295 (1.529-7.303)	0.004
No	1.000	
Posterior acoustic pattern		
Enhancement	1.596 (0.440-5.237)	0.319
Shadow	1.584 (0.634-3.910)	0.451
No change	1.000	

### The accuracy of SLNB in the determination of axillary lymph node tumor burden

**Figure 1:**
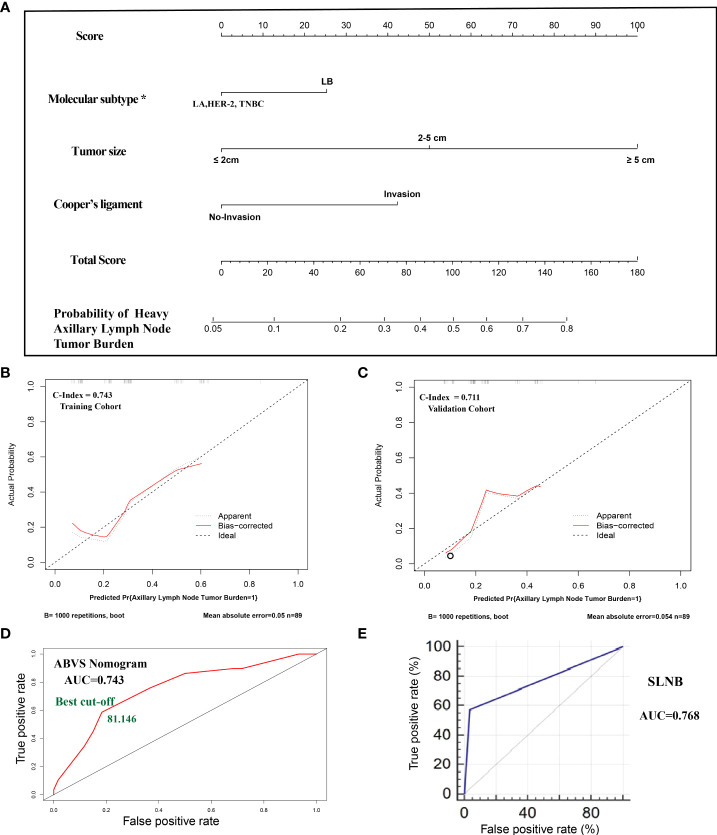
**(A)**. The nomogram clinical model. The predictors included tumor size, molecular classification, and Cooper’s ligament invasion. Among them, the molecular subtype of luminal B type was assigned a score of 25 points, and other molecular subtypes were assigned 0 points; the maximum lesion diameter of ≤2 cm was assigned 0 points, the maximum lesion diameter of 2–5 cm was assigned 50 points, and the maximum lesion diameter of ≥5 cm was assigned 100 points; the presence of the Cooper’s ligament invasion was assigned 42.5 points, and its absence was assigned 0 points. The probability of axillary lymph node tumor burden can be calculated after generating all of the assigned scores. **(B)**. The calibration of the training cohort. **(C)**. The calibration of the validation cohort. **(D)**. The ROC curve and best cutoff value of the nomogram clinical model. **(E)**. The AUC of sentinel lymph node biopsy. *LA: luminal A, LB: luminal B, TNBC: triple-negative breast cancer.

A total of 251 cases of breast lesions were included. One hundred forty-four cases underwent SLNB, 178 cases underwent ALND, and 71 cases underwent both operations. The 71 patients who underwent both SLNB and ALND were grouped according to the results of SLNB: 10 cases (14.085%) with heavy lymph node tumor burden and 61 cases (85.915%) with mild lymph node tumor burden. The results of SLNB were compared with the results of ALND, and the comparison showed a sensitivity of 57.143%, a specificity of 96.491%, and an accuracy of 88.732%. The graphed ROC curve is shown in **Figure 1E**, and the area under the ROC curve (AUC) is 0.768.

### Nomogram for predicting the probability of heavy lymph node tumor burden

All of the patients who underwent ALND were included in the cohort. The cohort was divided into a training set and a validation cohort at a 1:1 ratio in chronological order. A total of 178 cases of lesions were included, with 89 cases in each of the training sets and the validation cohort. The breast cancer preoperative examination indicators with statistical significance in the multivariate logistic analysis were included as predictors to establish a nomogram scoring system. The predictors included tumor size, molecular classification, and Cooper’s ligament invasion. Among them, the molecular subtype of luminal B type was assigned a score of 25 points, and other molecular subtypes were assigned 0 points; the maximum lesion diameter of ≤2 cm was assigned 0 points, the maximum lesion diameter of 2–5 cm was assigned 50 points, and the maximum lesion diameter of ≥5 cm was assigned 100 points; the presence of Cooper’s ligament invasion was assigned 42.5 points, and its absence was assigned 0 points. The statistical model automatically generated all of the assigned scores (**Figure 1A**). The concordance index (C-index) of the nomogram scoring system for predicting the probability of heavy lymph node tumor burden on the training set is 0.743, the average absolute error is 0.05 (**Figure 1B**), and the area under the curve is 0.743 (**Figure 1D**). The validation cohort was used to calibrate the nomogram scoring system for predicting the probability of heavy lymph node tumor burden. The calibration curve is shown in **Figure 1C** with a consistency index of 0.711 and an average absolute error of 0.054. The results of the validation set and the training set are consistent. The best cutoff value of the ABVS nomogram is 81.146 points according to the ROC curve.

**Figure 2:**
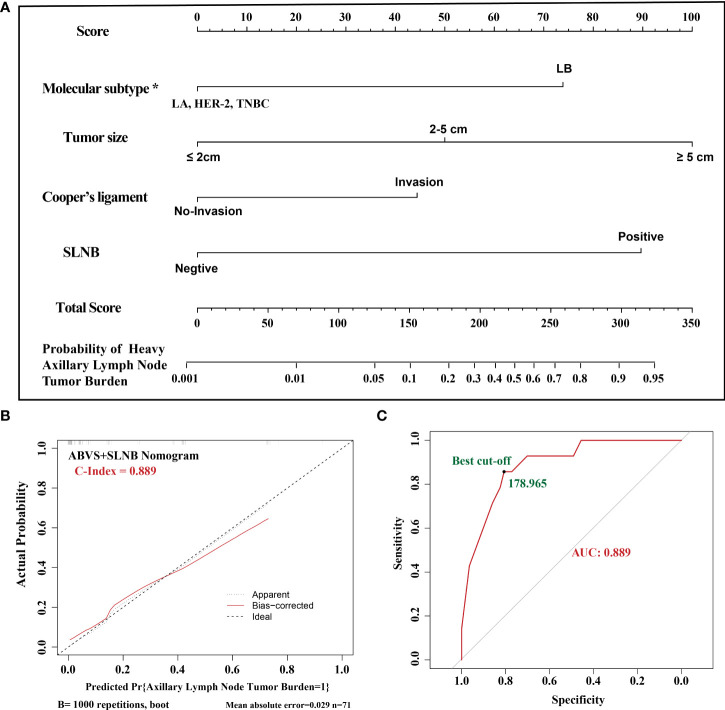
**(A)**. The ABVS and SLNB nomogram clinical model. The predictors included tumor size, molecular classification, and Cooper’s ligament invasion, SLNB. The probability of axillary lymph node tumor burden can be calculated after generating all of the assigned scores. **(B)**. The calibration. **(C)**. The ROC curve and best cutoff value. *LA: luminal A, LB: luminal B, TNBC: triple-negative breast cancer.

To confirm whether the ABVS nomogram can be a supplement to SLNB, we developed a new model based on ABVS features and SLNB. The results showed that the AUC and C-index are 0.889, and the average absolute error is 0.029. Meanwhile, the best cutoff value is 178.965 points according to the ROC curve (**Figure 2**).

## Discussion

In this study, we showed the landscape of ABVS features in breast cancer, including the analyses in different clinical subgroups and molecular subtypes. Then, we successfully identified tumor size and invasion of Cooper’s ligament as the lymph node tumor burden-related ABVS features, combined with the molecular subtype; we developed a nomogram prediction model, which has a convincible AUC (0.743), while the AUC of SLNB is 0.768. Furthermore, when in combination with ABVS and SLNB, the AUC can increase to 0.889. Therefore, this model may be used for individual decision-making.

In breast cancer, the expression status of ER, PR, and HER-2 has important predictive values for prognosis. The recurrence rate of ER- or PR-positive breast cancer changes with time (24–26). In this study, compared with other molecular subtypes of breast cancer, the luminal B type was more closely associated with heavy axillary lymph node tumor burden. Previously, it has been reported that poorly differentiated breast tumors are mainly of the luminal B type (27), and breast tumors with positive axillary lymph nodes are often of the luminal B type (27, 28). Luminal B-type breast cancer is more likely to have a heavier axillary lymph node tumor burden. This result may be due to the interaction of several steroid receptors. The plasminogen activator inhibitor is one of the predictors of axillary lymph node metastasis, but it only functions in PR-positive tumors (29). The expression of vimentin and Ki-67 may indicate that the long-term prognosis of ER-positive tumors is poor (27), and studies have shown that vimentin is positively correlated with the expression of ER in breast cancer (30, 31). Although whether the expression of ER and PR can be used as a predictor of axillary lymph node status is still controversial (32), there are studies suggesting the correlation between the expression status of ER and lymph node involvement (33). The expression level of Ki-67 can be used to measure the level of cell proliferation. Ki-67 <14% is considered a low proliferation state, and ≥14% is considered a high proliferation state (34). At present, the cutoff level of Ki-67 is still controversial (35, 36). Some studies suggested that using 20% as the cutoff value for Ki-67 could better reflect the proliferation status of tumor cells (37). Therefore, in the logistic regression analysis of this study, 20% was used as the cutoff value of Ki-67. The difference between luminal A-type and luminal B-type breast cancer lies in the different expression levels of Ki-67. The luminal B-type breast cancer has a higher expression level of Ki-67 than the luminal A type, and then the proliferation of its tumor cells is more active.

A study has shown that tumor size is one of the predictors of axillary lymph node metastasis (38). Some scholars have identified a linear relationship between tumor size and axillary lymph node metastasis (39). There were 20 cases with a maximum lesion diameter of ≥5 cm in this study. In this group, the risk of heavy axillary lymph node tumor burden was eight times the risk in other groups, which is basically consistent with the results of a previous study (5). The Cooper’s ligament is a fiber bundle between the breast’s lobules that connects the deep and top layer of thesuperficial fascia and supports and secures the breast. When the lesion invades the Cooper’s ligament, the ultrasound manifests traction and thickening of the Cooper’s ligament. In this study, according to whether the Cooper’s ligament was invaded, all of the patients were divided into two groups. The results showed that the risk of heavy axillary lymph node tumor burden when the Cooper’s ligament was invaded was three times higher than that of the non-invaded group, which is consistent with previous studies (40, 41). Neoadjuvant chemotherapy has a positive effect on prolonging the survival time of breast cancer patients; however, some studies have also shown that neoadjuvant chemotherapy cannot achieve the expected effect for all breast cancer patients (42). Neoadjuvant chemotherapy is not effective on lymph nodes, the efficacy of complete remission is only about 40%, and different molecular subtypes respond differently to neoadjuvant chemotherapy (23). Therefore, we included it in our multivariate analysis. The results showed that patients who received neoadjuvant chemotherapy had a higher lymph node tumor burden. The reason is that patients with late-stage cancer were included in neoadjuvant chemotherapy. At the same time, the effective rate of the treatment was low, and the response of lymph nodes was even lower, which led to false-positive results. Therefore, this result’s essential cause is that these patients were in an advanced stage and not because neoadjuvant chemotherapy aggravated lymph node metastasis.

Studies have suggested that pathological classification is one of the prognostic factors of breast cancer (43), but no significant statistical difference was found in this study. The possible reason may be that there is no linear correlation between the pathological classification and the malignant degree of breast cancer. The evaluation index of this study was lymph node tumor burden, i.e., classifying the degree of lymphatic metastasis instead of analyzing whether there is axillary lymph node metastasis in breast cancer, which may have caused indistinguishable pathological classification.

In the malignant and benign breast lesion differentiation, ABVS diagnostic performance is similar to that of handheld ultrasound (HHUS), based on the evidence available in the previous studies (8, 44, 45). However, a great advantage of ABVS in breast lesion characterization in comparison to HHUS is its capability of obtaining details on the reconstructed coronal plane’s morphological features (8). Therefore, it can be sensibly concluded that in terms of differential findings assisted by coronal reconstruction, ABVS might be better when compared to HHUS (8). In our analysis, the Cooper’s ligament has been confirmed to have a relation with lymph node tumor burden, owing to the sensibly and completely ability of ABVS. On the other hand, in thedifferentiation of breast lesions that are malignant and benign, the ABVS coronal plane retraction phenomenon is perceived as having high probability as a diagnostic feature. However, we have not found any reports exploring the relationship between retraction phenomenon and lymphatic metastasis of breast cancer. Our findings suggest that the retraction phenomenon may not be closely related to the lymphatic metastasis of breast cancer, and further verification is needed.

This study has limitations. This research is a retrospective study. All of the acoustic features of breast lesions were extracted from saved images. Although the saved images can be reconstructed by the workstation and viewed repeatedly, there are still possible information omissions or misjudgments. Some breast cancer lesions would not be identified well by sonography; therefore, the ABVS model may not be suitable for all the breast cancer patients, and more studies focusing on these patients are needed. However, the ABVS and SLNB model may be the solution for these patients; further studies are needed.

In conclusion, by integrating the real-world data, we showed the landscape of ABVS features in the breast cancer, including the analyses in different clinical subgroups and molecular subtypes. Then, we successfully identified the lymph node tumor burden-related ABVS features, combined with the molecular subtype, and we developed a nomogram prediction model, which may be used for individual decision-making in the diagnosis and treatment of breast cancer.

## Data availability statement

The original contributions presented in the study are included in the article/**Supplementary Material**. Further inquiries can be directed to the corresponding author.

## Ethics statement

The studies involving human participants were reviewed and approved by the ethics committee of Third Xiangya Hospital of Central South University. The patients/participants provided their written informed consent to participate in this study.

## Author contributions

FZ, CC, and JX designed the study. CC, FZ, and ML collected the data and performed the major analysis. JX supervised the study. CC and FZ analyzed and interpreted the data. CC and FZ did the statistical analysis. CC and FZ drafted the manuscript. All authors read and approved the final manuscript.

## Funding

This study was supported by Hunan Provincial Natural Science Foundation of China (No. 2019JJ40459) and the project of Health Commission of Hunan Province (No. B2019177).

## Conflict of interest

The authors declare that the research was conducted in the absence of any commercial or financial relationships that could be construed as a potential conflict of interest.

## Publisher’s note

All claims expressed in this article are solely those of the authors and do not necessarily represent those of their affiliated organizations, or those of the publisher, the editors and the reviewers. Any product that may be evaluated in this article, or claim that may be made by its manufacturer, is not guaranteed or endorsed by the publisher.
