# Supplementary material for: Identification of the lymph node metastasis-related automated breast volume scanning features for predicting axillary lymph node tumor burden of invasive breast cancer via a clinical prediction model
Source: Front Endocrinol (Lausanne). 2022 Aug 5;13:881761. doi: 10.3389/fendo.2022.881761 (PMC9388849; doi:10.3389/fendo.2022.881761)
Supplement: Supplementary file 1 [file Table_1.docx]

**Supplementary materials**

Table S1. The clinical feathers of 245 patients (251 tumor lesions)

| Clinical Feathers | | N | % |
| --- | --- | --- | --- |
| Age,y | ≤50 | 126 | 51.429 |
|  | >50 | 119 | 48.571 |
| Menopause | Yes | 106 | 43.265 |
|  | No | 139 | 56.735 |
| Tumor site | Left | 130 | 51.793 |
|  | Right | 121 | 48.207 |
| Pathologic type | Intraductal carcinoma in situ | 45 | 17.928 |
|  | Invasive ductal carcinoma | 185 | 73.705 |
|  | Invasive lobular carcinoma | 9 | 3.586 |
|  | Others | 12 | 4.781 |
| Molecular subtype | Luminal A | 58 | 23.108 |
|  | Luminal B | 124 | 49.402 |
|  | HER-2 | 35 | 13.944 |
|  | Triple negative | 34 | 13.546 |

Table S2. Relationship between the clinical features of breast cancer and sentinel lymph node tumor burden

| Variable | OR（95%CI ） | P |
| --- | --- | --- |
| Age, y |  |  |
| >50 | 0.774（0.191-2.833） | 0.702 |
| ≤50 | 1.000 |  |
| Molecular subtype |  |  |
| Luminal A | 0.415（0.016-10.889） | 0.542 |
| Luminal B | 2.267（0.377-43.556） | 0.455 |
| Triple negative | 0.000（0.000-1.115） | 0.992 |
| HER-2 | 1.000 |  |
| Ki67 |  |  |
| ≥20% | 4.100（0.735-76.852） | 0.188 |
| <20% | 1.000 |  |
| Neoadjuvant chemotherapy |  |  |
| Yes | 0.000（0.000-1.729） | 0.992 |
| No | 1.000 |  |
| Menopause |  |  |
| Yes | 0.676（0.141-2.550） | 0.582 |
| No | 1.000 |  |
| Tumor site |  |  |
| Left | 0.557（0.137-2.039） | 0.381 |
| Right | 1.000 |  |
| Nipple invasion |  |  |
| Yes | 0.000（0.000-2.789） | 0.993 |
| No | 1.000 |  |

Table S3. Relationship between the ABVS features of breast cancer and sentinel lymph node tumor burden

| Variable | OR（95%CI ） | P |
| --- | --- | --- |
| Tumor Size |  |  |
| ≥5cm | 6.500（0.701-60.974） | 0.080 |
| 2-5cm | 2.108（0.465-14.776） | 0.373 |
| ≤2cm | 1.000 |  |
| Orientation |  |  |
| Not Parallel | 0.494（0.073-2.074） | 0.385 |
| Parallel | 1.000 |  |
| Shape |  |  |
| Irregular | 9.885（0.000-9.969） | 0.992 |
| Regular | 1.000 |  |
| Margin |  |  |
| Spiculated | 3.429（0.000-4.571） | 0.996 |
| Microlobulated | 1.000（0.000-3,779） | 1.000 |
| Angular | 2.466（0.000-3.427） | 0.996 |
| Circumscribed | 1.000 |  |
| Echo Pattern |  |  |
| Hypoechoic | 9.718（0.000-9.844） | 0.992 |
| Mixed solid echo | 1.000 |  |
| Posterior acoustic pattern |  |  |
| Enhancement | 0.584（0.030-3.531） | 0.624 |
| Shadow | 1.169（0.166-5.246） | 0.852 |
| No change | 1.000 |  |
| Microcalcifications |  |  |
| Present | 0.676（0.141-2.550） | 0.582 |
| Absent | 1.000 |  |
| Acoustic halo |  |  |
| Present | 0.946（0.138-4.040） | 0.946 |
| Absent | 1.000 |  |
| Retraction phenomenon |  |  |
| Present | 0.765（0.040-4.461） | 0.805 |
| Absent | 1.000 |  |
| Invasion of Cooper’s ligament |  |  |
| Yes | 1.552（0.319-5.971） | 0.542 |
| No | 1.000 |  |
| BI-RADS |  |  |
| 5 | 1.020（0.000-2.395） | 0.993 |
| 4c | 1.285（0.000-2.412） | 0.993 |
| 4b | 5.641（0.000-6.439） | 0.994 |
| 4a | 9.252（0.000-9.817） | 0.993 |
| 3 | 1.000 |  |

Table S4. univariate-logistic regression analysis of the clinical features and axillary lymph node tumor burden

| Variable | OR（95%CI ） | P |
| --- | --- | --- |
| Age,y |  |  |
| >50 | 1.382（0.720-2.679） | 0.333 |
| ≤50 | 1.000 |  |
| Molecular subtype |  |  |
| Luminal A | 0.769（0.058-0.528） | 0.732 |
| Luminal B | 2.846（0.986-10.344） | 0.074 |
| Triple negative | 1.875（0.457-8.435） | 0.388 |
| HER-2 | 1.000 |  |
| Ki67 |  |  |
| ≥20% | 3.025（1.101-10.683） | 0.050 |
| <20% | 1.000 |  |
| Neoadjuvant chemotherapy |  |  |
| Yes | 4.181（1.509-12.202） | 0.006 |
| No | 1.000 |  |
| Menopause |  |  |
| Yes | 1.202（0.623-2.311） | 0.581 |
| No | 1.000 |  |
| Tumor site |  |  |
| Left | 0.747（0.385-1.433） | 0.382 |
| Right | 1.000 |  |
| Nipple invasion |  |  |
| Present | 6.793（1.411-48.598） | 0.025 |
| Absent | 1.000 |  |

Table S5. univariate-logistic regression analysis of the ABVS features and axillary lymph node tumor burden

| Variable | OR（95%CI ） | | P |
| --- | --- | --- | --- |
| Tumor Size |  | |  |
| ≥5cm | 5.128（1.530-18.232） | | 0.009 |
| 2-5cm | 1.625（0.784-3.510） | | 0.201 |
| ≤2cm | 1.000 | |  |
| Orientation |  | |  |
| Not Parallel | 0.823（0.409-1.615） | | 0.576 |
| Parallel | 1.000 | |  |
| Shape |  | |  |
| Irregular | 1.670（0.503-7.565） | | 0.443 |
| Regular | 1.000 | |  |
| Margin |  | |  |
| Spiculated | 3.275（0.000-3.344） | | 0.986 |
| Microlobulated | 8.636（0.000-9.625） | | 0.987 |
| Angular | 1.877（0.000-2.087） | | 0.986 |
| Circumscribed | 1.000 | |  |
| Echo Pattern |  | |  |
| Hypoechoic | 1.869（0.461-12.548） | | 0.435 |
| Mixed solid echo | 1.000 | |  |
| Posterior acoustic pattern |  | |  |
| Enhancement | 0.667（0.208-1.808） | | 0.454 |
| Shadow | 2.036（0.938-4.386） | | 0.069 |
| No change | 1.000 | |  |
| Microcalcifications | |  | |
| Present | 0.629（0.316-1.220） | | 0.176 |
| Absent | 1.000 | |  |
| Acoustic halo |  | |  |
| Present | 2.603（1.242-5.446） | | 0.011 |
| Absent | 1.000 | |  |
| Retraction phenomenon |  | |  |
| Present | 0.995（0.423-2.205） | | 0.991 |
| Absent | 1.000 | |  |
| Invasion of Cooper’s ligament | | |  |
| Yes | 3.206（1.645-6.353） | | 0.001 |
| No | 1.000 | |  |
| BI-RADS |  | |  |
| 5 | 3.000（0.649-15.398） | | 0.167 |
| 4c | 2.154（0.680-8.285） | | 0.219 |
| 4b | 1.458（0.402-6.116） | | 0.579 |
| 4a | 0.570（0.148-2.438） | | 0.421 |
| 3 | 1.000 | |  |
